# The REFANI-N study protocol: a cluster-randomised controlled trial of the effectiveness and cost-effectiveness of early initiation and longer duration of emergency/seasonal unconditional cash transfers for the prevention of acute malnutrition among children, 6–59 months, in Tahoua, Niger

**DOI:** 10.1186/s12889-015-2640-2

**Published:** 2015-12-23

**Authors:** Victoria L. Sibson, Carlos S. Grijalva-Eternod, Leila Bourahla, Hassan Haghparast-Bidgoli, Joanna Morrison, Chloe Puett, Lani Trenouth, Andrew Seal

**Affiliations:** UCL Institute for Global Health, London, WC1N 1EH UK; Concern Worldwide, Niamey, Niger; Action Against Hunger, New York, USA

**Keywords:** Acute malnutrition, Wasting, Cash transfer, Niger, Study protocol, Cost-effectiveness analysis

## Abstract

**Background:**

The global burden of acute malnutrition among children remains high, and prevalence rates are highest in humanitarian contexts such as Niger. Unconditional cash transfers are increasingly used to prevent acute malnutrition in emergencies but lack a strong evidence base. In Niger, non-governmental organisations give unconditional cash transfers to the poorest households from June to September; the ‘hunger gap’. However, rising admissions to feeding programmes from March/April suggest the intervention may be late.

**Methods/design:**

This cluster-randomised controlled trial will compare two types of unconditional cash transfer for ‘very poor’ households in ‘vulnerable’ villages defined and identified by the implementing organisation. 3,500 children (6–59 months) and 2,500 women (15–49 years) will be recruited exhaustively from households targeted for cash and from a random sample of non-recipient households in 40 villages in Tahoua district. Clusters of villages with a common cash distribution point will be assigned to either a control group which will receive the standard intervention (*n* = 10), or a modified intervention group (*n* = 10). The standard intervention is 32,500 FCFA/month for 4 months, June to September, given cash-in-hand to female representatives of ‘very poor’ households. The modified intervention is 21,500 FCFA/month for 5 months, April, May, July, August, September, and 22,500 FCFA in June, providing the same total amount. In both arms the recipient women attend an education session, women and children are screened and referred for acute malnutrition treatment, and the households receive nutrition supplements for children 6–23 months and pregnant and lactating women. The trial will evaluate whether the modified unconditional cash transfer leads to a reduction in acute malnutrition among children 6–59 months old compared to the standard intervention. The sample size provides power to detect a 5 percentage point difference in prevalence of acute malnutrition between trial arms. Quantitative and qualitative process evaluation data will be prospectively collected and programme costs will be collected and cost-effectiveness ratios calculated.

**Discussion:**

This randomised study design with a concurrent process evaluation will provide evidence on the effectiveness and cost-effectiveness of earlier initiation of seasonal unconditional cash transfer for the prevention of acute malnutrition, which will be generalisable to similar humanitarian situations.

**Trial registration:**

ISRCTN25360839, registered March 19, 2015.

## Background

Over one fifth of countries have a prevalence of acute malnutrition^1^ of 10 % or more, which is classified as “a public health emergency requiring immediate intervention” [1]. Humanitarian disasters may exacerbate pre-existing causes of undernutrition or create new risks, causing the prevalence of acute malnutrition to rise. Evidence exists on the effectiveness of food-based interventions for the treatment of severe acute malnutrition and moderate acute malnutrition [2], but their coverage is low [3] and costs are high [4].

Preventative interventions are also necessary and a range of sectoral activities may be required to tackle the multiple potential causes of undernutrition, including insufficiencies in household food security and diet, challenges in the social and care environment, and disease and a poor public health environment. Amongst these potential interventions, food-based approaches such as General Food Distributions (GFD) and Blanket Supplementary Feeding Programmes (BSFP) predominate. There is, however, limited evidence of their positive impact [5], critical challenges associated with their implementation [6, 7] and concerns about their cost-effectiveness [8].

As a consequence of the limitations of food aid, other forms of food assistance, such as short term cash and voucher interventions, have become more commonly used to try and prevent acute malnutrition in emergency situations in recent years [9]. But, whilst cash/vouchers have the potential to address various causes of undernutrition [10], there is limited and inconsistent evidence on their nutritional impact and little knowledge about which of the impact pathways may be influenced in different contexts.

Studies have shown that cash or vouchers can impact positively on diets and food security at a household level [11], however, little is known about their impact at an individual level. Similarly, evidence from longer term conditional cash transfers has shown that they can improve uptake of health services where services are available. However, this improved care-seeking does not often translate into improved health or nutrition outcomes [11].

Furthermore, it is generally agreed that implementation of cash/voucher programmes as stand-alone interventions are less likely to be efficacious, regardless of the different pathways by which they may impact nutrition status [12–14]. Understanding when cash and vouchers can work to reduce malnutrition and when and what other complementary interventions are required for effectiveness remains a research priority.

Seasonal Unconditional Cash Transfers (UCT) have been implemented by non-governmental organisations (NGO) in Niger since 2004 as a part of humanitarian programming. However, studies or evaluations undertaken to assess their nutritional impact have yielded inconsistent and inconclusive results [15–22]. Questions on the most effective timing, amount, and duration remain unanswered. It has become common practice to deliver cash from June to September to coincide with the annual ‘hunger gap’. However, monthly trends in the admission of acutely malnourished children to feeding programmes indicate that changes in incidence may not coincide with this lean season, with admission rising from March/April and again in September. There is also an absence of evidence on the impact of this economically targeted intervention at the population level.

**Fig. 1:**
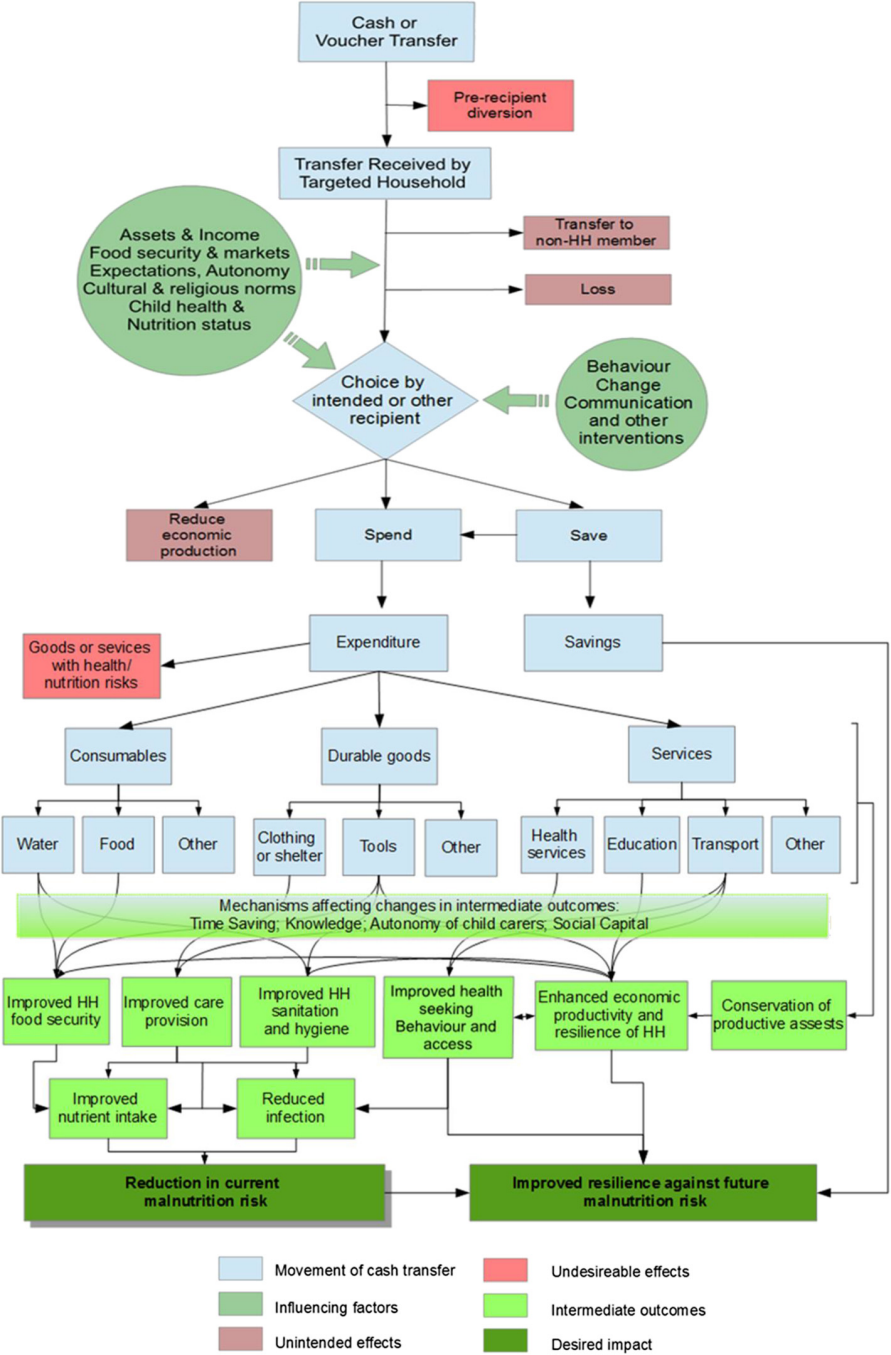
The REFANI studies theory of change

This study aims to assess, on both targeted households and at the population level, the nutritional impact of a modified UCT that starts earlier in the year. It also aims to explore the pathways, shown in the REFANI theory of change (Fig. 1), by which the intervention may work, through changes in food security or other pathways. Lastly, the study will add to the sparse evidence base on the cost and cost effectiveness of cash transfer interventions. It is one of three studies undertaken by the REFANI consortium (the other two being in Pakistan and Somalia) with the common objective of enhancing understanding of the pathways by which cash and voucher transfers may work to reduce the risk of acute malnutrition in humanitarian emergencies.

## Methods/design

### Objectives

To conduct a cluster randomised controlled trial (cRCT) of the effectiveness and cost-effectiveness of an earlier and longer (April-September) seasonal UCT intervention for preventing acute malnutrition in children 6–59 months of age, compared with the standard (June-September) seasonal UCT. Both UCT will be of equal total value and will target ‘very poor’ households in the Tahoua district of rural Niger. We will also conduct a process evaluation to describe the context and intervention implementation, and to explore pathways by which the intervention works or fails to work. This will inform judgements on the generalisability of the findings to other humanitarian contexts where cash transfers are being used to prevent acute malnutrition.

### Hypotheses

We have three hypotheses. 1. An earlier seasonal UCT, of equal value and longer duration than the standard intervention, reduces the prevalence of acute malnutrition in children aged 6–59 months, in the targeted ‘very poor’ households by the end of the lean season. 2. An earlier seasonal UCT, of equal value and longer duration than the standard intervention, reduces the prevalence of acute malnutrition in children, 6–59 months of age, in the communities in which the cash recipients live, by the end of the lean season. 3. The earlier seasonal UCT of equal total value to the standard intervention is more cost-effective per case of acute malnutrition averted compared to the standard intervention.

### Study setting and population

The study will be implemented in the communes of Affala and Takanamatt in the district of Tahoua in south west Niger where the NGO Concern Worldwide has been implementing multi-sector development and humanitarian assistance programmes since 2003. The majority ethnic group is Hausa, followed by Tuareg and Fulani. Agro-pastoral livelihoods and sedentary lifestyles predominate. The patriarchal culture is often associated with female disempowerment where male household heads are the main decision makers and control the majority of the household’s resources [23]. Literacy rates are very low, particularly among women [24].

There is a single short and unpredictable rainy season between June and September, which is also considered the ‘lean season’, which provides rain fed irrigation for millet, the staple crop. However, most households are unable to produce sufficient cereals for their own consumption, even in ‘normal years’ [25]. Households rely heavily on daily wage labour, labour migration, petty trade, taking credit and selling assets to maintain food access [25]. Besides food insecurity, challenges in the public health and social and caring environment, which are known risk factors for malnutrition, also exist. The environment is arid with a chronic lack of water, low coverage of latrines, and poor hygiene and sanitation practices are common [23]. Malaria is endemic and diarrhoea and acute respiratory infection rates are also high [23]. Despite the existence of free health care for children under five and pregnant women, geographic inaccessibility and the poor quality of the formal health service leads to low utilisation.

As a consequence of these challenges, nutrition surveys indicate that acute malnutrition prevalence 2 is persistently above 10 % in July, during the lean season, and is similar in the post-rains, post-harvest period in December. As well as working with government health services to treat children with acute malnutrition, Concern Worldwide implements multisector programmes to address the causes of undernutrition. These include seasonal cash transfers.

### Interventions

The selection of households that will be eligible to receive one of two UCT interventions will take place in a two stage process that is undertaken by Concern Worldwide as part of their normal humanitarian programming. First, villages will be targeted for UCT interventions based on their perceived vulnerability to food insecurity. Then, within the targeted villages, a wealth ranking is assigned to households using a household survey approach.^3^ This draws on the principles of HEA and has been standardised among NGOs in Niger [26]. Household with a very poor ranking are targeted for cash transfers.

The control intervention is a standard UCT package delivered by Concern Worldwide in Niger as a part of their humanitarian programmes. Each selected household receives 32,500 FCFA^4^ a month, which is given in cash to female representatives who must attend a cash distribution point every month, from June to September. Cash distribution points are sited in villages so that beneficiaries do not have to travel more than 5 km. The total seasonal cash transfer is 130,000 FCFA. The cash amount is calculated by the government to allow purchase of a food basket similar to the World Food Programme (WFP) household ration that will meet 75 % of the daily kilocalorie needs of a household of seven people. Cash is given to women on the assumption that they will be able to influence its use to the benefit of children. The cash transfer is manual because the mobile phone network does not support money transfers. A nutrition supplement is given to any pregnant and lactating women (PLW) and children 6–23 months old in the cash receiving household regardless of their nutritional status, as long as they attend on the distribution day. At the distribution, women must attend a health, hygiene, and nutrition education session and PLW and children are screened for acute malnutrition using Mid Upper Arm Circumference (MUAC) and referred to supplementary or therapeutic feeding programmes according to referral criteria. Concern Worldwide also undertakes community sensitisation on the objectives of the cash programme, including suggestions to use the cash to buy food for children.

**Fig. 2:**
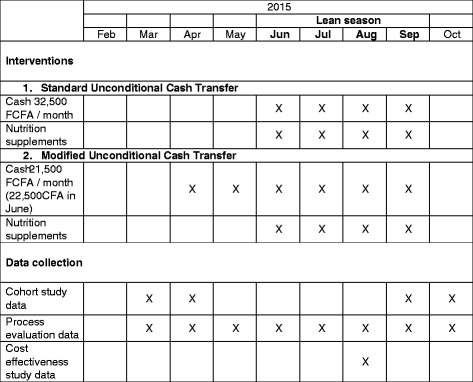
REFANI-N intervention and data collection schedule

The modified intervention gives the same total amount of cash to households, but over a longer period of time. A UCT of 21,500 FCFA is given manually to female household representatives who must attend a cash distribution point every month from April to September (22,500 FCFA is given in June). The total seasonal cash transfer is the same as for the standard intervention (130,000 FCFA). The intervention is the same as the standard intervention in all other ways and nutrition supplements are only given between June and September. Figure 2 illustrates the intervention components and timings, alongside the data collection plans.

### Study design and randomisation

The study design is a community-based, two-arm cluster RCT. We will assess the impact of UCT interventions on the risk of child undernutrition among households targeted for intervention and also in the community in which the interventions are implemented. A clustered design is necessary to ensure feasibility of delivery of the interventions being tested and to prevent contamination. The unit of randomisation will be the cash distribution point; i.e. one or more villages grouped to receive cash in a given location. Households will be assigned to the trial arm on the basis of their village of residence. The randomisation will be undertaken in a public meeting to which the village leaders will be invited. The names of the villages and their cash distribution point will be written on pieces of paper placed in sealed envelopes of identical shape size and colour. Meeting participants will be invited to blindly select envelopes one by one which will be sequentially allocated to the modified and standard cash arms, until all have been allocated. There will be around 20 cash distribution points, which will result in each intervention group having 10 clusters that will contain around 20 villages.

Cash receiving households will be exhaustively sampled from the selected villages for inclusion in the study cohort. In addition, a sample of households not eligible for cash transfers will be randomly sampled for inclusion in the cohort, from the same villages. The study participants will be children 6–59 months and women 15–49 years, exhaustively sampled from the selected households. The target number of participants is 3,500 children (6–59 months) and 2,500 women (15–49 years). Disabled and seriously sick individuals will be excluded.

Questions related to household costs and income will be included in the endline cohort questionnaire. For collection of qualitative data on household and community costs, up to 10 villages will be selected purposively using selection criteria (e.g. village size, whether village is located at a distribution point, remoteness of village, receipt of standard or modified intervention) to ensure a wide range of village characteristics are represented in the sample. Up to nine participants for the focus group discussions will then be selected randomly from amongst the programme beneficiaries in each village.

### Participant recruitment and informed consent

Informed verbal consent will be obtained from village leaders and representatives for household level data collection. At the household level, fieldworkers will provide information about the study objectives and data collection requirements, including how long the survey will take, the measurements needed and potential risks, to each individual in Hausa or their preferred language. Informed written consent will then be obtained from the head of household or another adult representative, following which informed written consent will be obtained from each adult participating in the study and from an adult for each child participating in the study. Each individual must provide a signature or written mark on the consent form to prove their willingness to participate. Households and individuals will not be pressurised to participate and it will be made clear that participation or non-participation will not affect their entitlement to humanitarian or any other forms of assistance. Participants will also be given general information about the interventions to which they have been allocated or an explanation if they have not been targeted, with advice to contact Concern Worldwide staff for more information if required.

For the focus group discussions for the cost-effectiveness analysis, a standardised text will be read aloud and informed verbal consent will be obtained from participants at the start of each discussion. It will be made clear that their participation and any information they volunteer will not affect their eligibility for the ongoing programme nor any potential future programmes. For key informant interviews, the purpose of the study will be explained prior to starting the interview and informed verbal consent will be obtained.

### Data collection

Data collection plans are illustrated in Fig. 2. Quantitative data collection from the cohort will take place at two time points, in March-April before the intervention and after the intervention in September-October. Trained staff will collect data under the supervision of a study coordinator and field manager. We will use structured questionnaires administered to the head of household, women of childbearing age (15–49 years) and mothers or carers of eligible children. Questionnaire tools will be developed using standard indicators, and refined based on the results of formative research and piloting. Data will be collected using Digital Data Gathering devices.

Qualitative data collection to describe the context and explore mechanisms by which the interventions work or fail to work will involve a longitudinal study of a purposively selected sample of cash receiving and non-cash receiving households, and additional, topic-focused qualitative studies. Data will be collected using interviews, focus group discussions and observation notes. Interviews and discussions will be recorded and a purposive sample transcribed and translated. Researchers will write descriptive reports of data that is not transcribed. The compilation of routine quantitative monitoring data, collected by Concern Worldwide during the intervention period from April to September, will help us to describe the study context and how this changes over time, and will allow for evaluation of the interventions’ implementation.

For cost-effectiveness analysis, institutional costs will be assessed using accounting data and information collected from key informant interviews and programme documentation. Societal and households costs will be assessed using qualitative data collected from focus group discussions and quantitative data collected from the cohort. Data collection for the cost-effectiveness analysis will be conducted by a separate study team including an international researcher and a translator, and a note-taker during community-level discussions.

### Outcome measures

The primary outcome measure is the prevalence of global acute malnutrition (weight for height < −2 Z-scores (WHO 2006 growth standards) and/or nutritional oedema) in children aged 6–59 months. Secondary outcomes measures are 1. Prevalence of global acute malnutrition (MUAC <12.5 cm and or nutritional oedema in children 6–59 months), 2. Mean weight for height (WHO 2006 growth standards) in children 6–59 months, 3. Mean MUAC in children 6–59 months, 4. Mean haemoglobin concentration in children 6–59 months, 5. Prevalence of anaemia in children 6–59 months, 6. Mean haemoglobin concentration in women aged 15–49 years, 6. Prevalence of anaemia in women aged 15–49 years, 8. Household Dietary Diversity Score, 9. Individual Dietary Diversity Score in children 6–59 months, 10. Individual Dietary Diversity Score in women 15–49 years, 11. Household expenditure and multidimensional poverty, 12. Cost effectiveness of the modified cash intervention.

### Measurement instruments

The weight of the children and women will be measured using an electronic scale measuring to 0.1 kg precision. Length (for children <24 months of age) and height (for children ≥24 months of age and women) will be measured using a baby mat and/or stadiometer measuring to 0.1 cm precision. MUAC will be measured on the left arm using an insertion tape to 0.1 cm precision. Bilateral pitting oedema will be assessed through applying medium pressure on the dorsum of both feet with thumbs for three seconds and determining presence/absence of an indentation. Haemoglobin concentration will be measured using the HemoCue 301 analyser to 0.1 g/dL precision.

### Standardisation procedure

Enumerators will be trained in anthropometric measurements and standardisation tests will be undertaken to assess the precision and accuracy of their measurements. Plausibility checks will be run on each team’s data using the software Emergency Nutrition Assessment [27], to examine the quality of the data and determine need for intensive retraining and closer supervision. Digital scales will be checked for accuracy daily using known weights. Structured questionnaires will be standardised and piloted prior to finalisation. Training of enumerators will ensure complete understanding of the questions in French and their appropriate translation in to Hausa. Qualitative fieldworkers will be hired for their skills and experience in collecting qualitative data and will be trained and supervised by an experienced qualitative researcher.

### Sample size calculation

In order to detect a difference of 5 percentage points in the prevalence of acute malnutrition among children 6–59 months of age between the trial arms, assuming a fixed number of 20 clusters and end-line prevalence of 21 %, and allowing a type 1 error rate of 5 % and power of 80 %, we calculated that a minimum sample size of 1,767 children in 10 clusters for each arm will be necessary. Because the clustered sample design will induce correlation within sampling units, we used a sample size calculation method designed for use with clustered RCTs [28]. We also accounted for variable numbers of children served by each distribution point in calculating our final sample size.

### Data analyses

We will test for an end-line difference in the prevalence of acute malnutrition, among children 6–59 months, between the cash receiving households in the two study arms. Analysis will be conducted using generalized estimating equations (GEE) and adjusted for correlation of responses within clusters. We will choose our covariates and identify potential confounders using the pathways described in the theory of change (Fig. 1), and explore the intervention’s mechanisms of action. We will also undertake post hoc analyses by age strata, <24 months and ≥24 months. We will also test for an end-line difference in acute malnutrition prevalence between non-beneficiary households in the two arms of the trial, to assess the impact of the modified intervention at a population level.

Formative research data about context will be synthesized into a narrative report, which will be updated as contextual changes occur. Qualitative data from the longitudinal and focused studies will be analysed using descriptive content analysis, comparing views from different participants within each intervention group in the trial and members of non-cash receiving households to assess the extent of triangulation and explore how the intervention receipt affected different participants. Researchers will discuss emergent themes, and coding structures will be developed from these themes and from the research questions. Data will then be coded in NVivo V10. Data will be summarised and described using illustrative quotes. Longitudinal qualitative data collection will be analysed between data collection points, to build our theory of how the intervention is working or not working. We will discuss and validate the development of our theory of change and research findings with a sample of research participants and team members at two time points, one during the intervention implementation, and one after the UCT has ceased.

Cost-effectiveness analysis will combine quantitative and qualitative data on institutional, beneficiary, and community-level costs to estimate total intervention costs from a societal perspective. The analysis will be done using Microsoft Excel and TreeAge software packages. Community-level qualitative data will be analysed for themes related to the opportunity cost of participation and resources required to access the program. An activity-based cost analysis will be conducted, allocating and estimating costs per major program activity using staff time allocation proportions. Cost-effectiveness will be assessed using incremental cost-effectiveness ratios (ICER), representing the additional cost per successful outcome achieved (e.g. case of acute malnutrition averted) by the modified relative to the standard intervention. Additional costing metrics will be calculated, including cost per beneficiary and cost-transfer ratio. Sensitivity analyses will be conducted to gauge whether plausible changes in various study parameters would result in significant changes in the base case cost-effectiveness outcomes.

### Ethical considerations

The study protocol was approved by two independent ethics committees; the Comité Consultatif National d'Ethique at the Ministry of Health in Niger (ID number 021/2014/CCNE).and the University College London Research Ethics Committee (project ID 6543/001). This study is also registered with ISRCTN (ISRCTN25360839). Informed consent procedures described above will allow individual participants to decline to participate. Regardless of whether potential participants consent to take part in the study, the study teams will offer to screen women and children in the sampled households for acute malnutrition and anaemia and refer them to health facilities for treatment according to local diagnostic criteria.

Trial safety will be monitored by measuring MUAC in children brought to the cash distributions in June and July. Our stopping rule will be a significant difference of ≥5 percentage points in the prevalence of low MUAC between arms at either time point, with a *p* < 0.001. Analysis will be conducted using the Wald chi-square test in Stata 13, adjusted for cluster, age, and sex.

### Collaborating organisations

Concern Worldwide is implementing this study with technical direction from University College London and support from Action Against Hunger. It is funded by the UK Department for International Development. The cash intervention is implemented by Concern Worldwide who sub-contract a local microfinance institution called Asus to provide the cash, and nutrition supplements are provided by the UN World Food Programme. The UCT interventions are funded by the EU Humanitarian Aid and Civil Protection Department (ECHO).

## Discussion

Study implementation has been ongoing since March 2015. Some deviations from the protocol have occurred to maximise implementation feasibility in the field. Instead of a planned cross sectional survey, a sample of non-cash receiving households was included in the cohort study. Due to staffing challenges the longitudinal qualitative study has been changed to a before and after study and there will be limited focused thematic investigation. Whilst the cohort study is being implemented to the forecast timeline, the end of the process evaluation is now forecast to be in Spring 2016.

The strong study design presented here, with its randomisation of intervention clusters, will provide new and robust evidence on the effectiveness and cost effectiveness of extending emergency seasonal transfers over a longer period. The results will also provide new information on the population level impact of poverty targeted UCT, which does not yet exist. Data on context and intervention mechanisms collected through the process evaluation will provide evidence that will be generalisable to similar humanitarian situations.

## Abbreviations

BSFP: Blanket Supplementary Feeding Programme
ENA: Emergency Nutrition Assessment
FCFA: Franc of the Financial Community in Africa
GEE: Generalised Estimating Equations
GFD: General Food Distribution
MUAC: Mid Upper Arm Circumference
NGO: Non-Governmental Organisation
PLW: Pregnant and Lactating Women
UCT: Unconditional Cash Transfer
WHO: World Health Organisation
WHZ: Weight for Height Z-Score

## Footnotes

1 Defined as a weight for height Z-score (WHZ) < −2 (WHO 2006 growth standards) and/or nutritional oedema.

2 Proportion of children, aged 6-59 months, with a weight for height Z-score (WHZ) < −2 (WHO 2006 growth standards) and/or nutritional oedema.

3 Defined by Concern Worldwide as a group of people preparing food and eating together.

4 1000 FCFA = £1.10, making 32,500 FCFA equivalent to £35.66 (21/08/2015)

## References

[1] United Nations Children’s Fund, World Health Organization, The World Bank. UNICEFWHO-World Bank Joint Child Malnutrition Estimates. UNICEF, New York; WHO, Geneva; The World Bank, Washington, DC; 2012.

[2] Webb P (2015). How strong is our evidence for effective management of wasting? A review of systematic and other reviews. Food Nutr Bull.

[3] Rogers E, Myatt M, Woodhead S, Guerrero S, Alvarez JL (2015). Coverage of community-based management of severe acute malnutrition programmes in twenty-one countries, 2012–2013. PLoS One.

[4] Bhutta ZA, Das JK, Rizvi A, Gaffey MF, Walker N, Horton S (2013). Evidence-based interventions for improvement of maternal and child nutrition: what can be done and at what cost?. Lancet.

[5] Sguassero Y, de Onis M, Bonotti AM, Carroli G (2012). Community-based supplementary feeding for promoting the growth of children under five years of age in low and middle income countries. Cochrane Database Syst Rev.

[6] US Centers for Disease Control and Prevention Evaluation of a Blanket Supplementary Feeding Program in Two Counties in Kenya, August 2011 – March 2012. Atlanta, USA; 2013.

[7] Oriere M, Hall A, Ndumi A (2010). Evaluation of the Emergency Blanket Supplementary Feeding Programme in Five Districts of Northern Kenya Save the Children UK.

[8] Puett C, Salpeteur C, Lacroix E, Houngbe F, Ait-Aissa M, Israel A-D (2013). Protecting child health and nutrition status with ready-to-use food in addition to food assistance in urban Chad: a cost-effectiveness analysis. Cost effectiveness and resource allocation : C/E.

[9] Harvey P, Proudlock K, Clay E, Riley B, Jaspars S (2010). Food aid and Food Assistance in Emergency and Transitional Contexts: A Review of Current Thinking.

[10] Leroy JL, Ruel M, Verhofstadt E (2009). The impact of conditional cash transfer programmes on child nutrition: a review of evidence using a programme theory framework. J Develop Effect.

[11] Manley J, Gitter S, Slavchevska V (2013). How effective are cash transfers at improving nutritional status?. World Dev.

[12] Bailey S, Hedlund K (2012). The Impact of Cash Transfers on Nutrition in Emergency and Transitional Contexts: A Review of the Evidence.

[13] Holmes R, Bhuvanendrah D (2013). Social Protection and Resilient Food Systems: The Role of Cash Transfers.

[14] Ruel MT, Alderman H (2013). Maternal Child Nutr Study G. Nutrition-sensitive interventions and programmes: how can they help to accelerate progress in improving maternal and child nutrition?. Lancet.

[15] Fenn B, Noura G, Sibson V, Dolan C, Shoham J (2014). The role of unconditional cash transfers during a nutritional emergency in Maradi region, Niger: a pre-post intervention observational study. Public Health Nutr.

[16] Aker J (2011). Zap It to Me: The Short-Term Impacts of a Mobile Cash Transfer Program.

[17] Children S (2009). How Cash Transfers can Improve the Nutrition of the Poorest Children: Evaluation of a Pilot Safety net Project in Southern Niger Save the Children.

[18] Poulsen L, Fabre D (2011). UNICEF Emergency Project Niger: Cash Transfer for Protection of Blanket Feeding, Maradi and Tahoua Regions: Independent Final Evaluation.

[19] Langendorf C, Roederer T, de Pee S, Brown D, Doyon S, Mamaty AA (2014). Preventing acute malnutrition among young children in crises: a prospective intervention study in Niger. PLoS Med.

[20] Aker JC, Nene M. Cash Transfers, Nutrition and Household Well-Being in Niger. Concern Worldwide, 2012

[21] Bliss J, Golden K. The Impact of Cash Transfers on Dietary Practices and Nutritional Status of Children 6–23 Months of Age in the District of Tahoua, Niger. Cornell University and Concern Worldwide, Sciences DoN; 2013.

[22] Bliss J, Jensen N, Thiede B, Shoham J, Dolan C, Sibson V, et al. The risk of acute malnutrition among children aged 6-36 months in households targeted by an emergency cash transfer program in Niger. Faseb J. 2014;28(1). 10.1177/0379572116654772 27402641

[23] Hampshire K, Casiday R, Kilpatrick K, Panter-Brick C (2009). The social context of childcare practices and child malnutrition in Niger’s recent food crisis. Disasters.

[24] INS, International (2012). I. Enquête Démographique et de Santé et à Indicateurs Multiples du Niger 2012 : Rapport de synthèse.

[25] Anonymous. Profils de Moyens d’Existence : Niger Zone Agro-pastorale du département Tahoua. 2012. http://www.heawebsite.org/countries/niger/reports/hea-lz-profile-agropastoral-livelihood-zone-tahoua-department-niger-2012.

[26] Boudreau T, Lawrence M, Izmann P, O’Donnell M, Adams L, Holt J (2008). The Practitioners’ Guide to the Household Economy Approach: Food Economy Group.

[27] Jayasekaran D. SMART Emergency Nutrition Assessment for Stanardized Monitoring and Assessment of Relief and Transitions (ENA for SMART). Software User Manual 2012. Available from: http://smartmethodology.org/survey-planning-tools/smart-emergency-nutrition-assessment/. Accessed on 21/12/2015.

[28] Hemming K, Girling AJ, Sitch AJ, Marsh J, Lilford RJ (2011). Sample size calculations for cluster randomised controlled trials with a fixed number of clusters. BMC Med Res Methodol.

